# Effect of Web-Based Versus Paper-Based Questionnaires and Follow-Up Strategies on Participation Rates of Dutch Childhood Cancer Survivors: A Randomized Controlled Trial

**DOI:** 10.2196/cancer.3905

**Published:** 2015-11-24

**Authors:** Ellen Kilsdonk, Eline van Dulmen-den Broeder, Helena J van der Pal, Nynke Hollema, Leontien C Kremer, Marry M van den Heuvel-Eibrink, Flora E van Leeuwen, Monique W Jaspers, Marleen H van den Berg

**Affiliations:** ^1^ Centre for Human Factors Engineering of interactive Health Information Technology (HIT-lab) Department of Medical Informatics Academic Medical Center Amsterdam Netherlands; ^2^ Department of Pediatrics Division of Oncology-Hematology VU University Medical Center Amsterdam Netherlands; ^3^ Late Effects Outpatient Clinic for Adult Survivors of Childhood Cancer Department of Medical Oncology Academic Medical Center Amsterdam Netherlands; ^4^ Dutch Childhood Oncology Group – Late Effects Registry The Hague Netherlands; ^5^ Department of Pediatric Oncology Emma Children’s Hospital/Academic Medical Center Amsterdam Netherlands; ^6^ Department of Pediatric Oncology/Hematology Sophia Children’s Hospital/Erasmus Medical Center Rotterdam Netherlands; ^7^ Department of Epidemiology Netherlands Cancer Institute Amsterdam Netherlands

**Keywords:** childhood cancer survivors, follow-up strategies, participation rates, questionnaires, questionnaire mode

## Abstract

**Background:**

Questionnaires are widely used in survey research, especially in cohort studies. However, participation in questionnaire studies has been declining over the past decades. Because high participation rates are needed to limit the risk of selection bias and produce valid results, it is important to investigate invitation strategies which may improve participation.

**Objectives:**

The purpose of this study is to investigate the effect of Web-based versus paper-based questionnaires on participation rates in a questionnaire survey on late effects among childhood cancer survivors (CCSs).

**Methods:**

A total of 750 CCSs were randomized across 3 study arms. The initial invitation in study arms 1 and 2 consisted of a Web-based questionnaire only, whereas in study arm 3 this invitation was complemented with a paper-based version of the questionnaire. The first postal reminder, sent to the nonresponding CCSs in all 3 study arms, consisted of either a reminder letter only (study arms 1 and 3) or a reminder letter complemented with a paper-based questionnaire (study arm 2). The second postal reminder was restricted to CCSs in study arms 1 and 2, with only those in study arm 1 also receiving a paper-based questionnaire. CCSs in study arm 3 received a second reminder by telephone instead of by mail. In contrast to CCSs in study arm 3, CCSs in study arms 1 and 2 received a third reminder, this time by telephone.

Results*:* Overall, 58.1% (436/750) of the CCSs participated in the survey. Participation rates were equal in all 3 study arms with 57.4% (143/249) in arm 1, 60.6% (152/251) in arm 2, and 56.4% (141/250) in arm 3 (*P*=.09). Participation rates of CCSs who received an initial invitation for the Web-based questionnaire only and CCSs who received an invitation to complete either a paper-based or Web-based questionnaire did not differ (*P*=.55). After the first postal reminder, participation rates of CCSs invited for the Web-based questionnaire only also did not differ compared with CCSs invited for both the Web-based and paper-based questionnaires (*P*=.48). In general, CCSs preferred the paper-based over the Web-based questionnaire, and those completing the paper-based questionnaire were more often unemployed (*P*=.004) and lower educated (*P*<.001).

**Conclusion:**

Invitation strategies offering a Web-based questionnaire without a paper-based alternative at first invitation can be used without compromising participation rates of CCS. Offering the choice between paper- and Web-based questionnaires seems to result in the highest accrual participation rate. Future research should look into the quality of the data delivered by both questionnaires filled in by respondents themselves.

**Trial Registration:**

International Standard Randomized Controlled Trial Number (ISRCTN): 84711754; http://www.controlled-trials.com/ISRCTN84711754 (Archived by WebCite at http://www.webcitation.org/6c9ZB8paX)

## Introduction

Owing to better stratification and advances in treatment regimens, childhood cancer survival rates have substantially increased in recent decades, resulting in a growing absolute number of childhood cancer survivors (CCSs). At present, approximately 75-80% of patients are expected to survive at least 5 years postdiagnosis [1]. Unfortunately, childhood cancer and its treatment can significantly impair long-term health and cause substantial excess morbidity [2-4] and mortality [5-9] even many years after treatment.

To gain insight into the long-term outcomes of children with cancer, patient-reported outcomes collected by questionnaires are essential. In the Netherlands, the Dutch Childhood Oncology Group Late Effects Group (DCOG LATER) initiated the “DCOG LATER” study, a nationwide study investigating late effects among these patients. As part of this retrospective cohort study, CCSs will be asked to complete a general health and lifestyle questionnaire to identify late effects not yet recognized and to define CCSs groups at high risk of developing such late adverse effects. In such studies, high participation rates are crucial for the validity of the results [10]. However, participation rates in questionnaire studies have been declining over the past 30 years, mainly due to an increase in the proportion of individuals declining participation or not responding at all [11]. Several studies have shown that participation rates of CCSs invited to questionnaire studies vary between 50% and 90% [12-19].

One proven way of increasing participation rates is to use reminders by regular mail or telephone calls [20-23]. In addition, recent studies have shown that using paper-based as well as Web-based questionnaire modes in the same study might increase response rates [11,23,24]. Web-based questionnaires are preferred by investigators because there are no printing and mailing costs involved and the time spent on data entry is minimized [25]. However, it is known that participant characteristics may influence the questionnaire mode preferred by participants [11,23,25,26]. For example, men tend to respond to Web-based questionnaires at a higher rate than women [25,27] and older participants seem to prefer paper-based over Web-based questionnaires [11,27-29]. A mixed-mode rather than a single mode design may overcome these limitations by providing survivors the opportunity to choose their preferred mode [23].

Other childhood cancer survivor studies have raised concerns about selection bias due to nonparticipation of CCSs not suffering from late effects [7,10,30,31]. High participation rates are required to limit the risk of selection bias and increase statistical power. Therefore, it is important to evaluate which invitation strategy leads to the highest participation rate; however, current studies focused on strategies to improve participation of CCSs in questionnaire surveys are lacking. The purpose of this study is to answer 2 questions. The first is do participation rates of CCSs who are invited to complete a Web-based questionnaire only differ from CCSs who are invited to complete either a Web-based or paper-based questionnaire? And second, what is the effect of adding a paper-based questionnaire to a postal reminder on participation rates? In addition, the reasons for nonparticipation, differences in participants’ questionnaire mode preferences, and their satisfaction with the different questionnaire modes was addressed. In this trial, CCSs were randomized to 1 of 3 study arms with different questionnaire modes and reminders.

## Methods

### Eligible Population

This study was conducted in 3 of the 7 Dutch Pediatric Oncology Centers (EKZ/AMC Amsterdam, Erasmus MC Rotterdam, and VUmc Amsterdam). Ethical Review Board approval was obtained in each participating center. We randomly selected 750 adult CCSs from the DCOG LATER cohort, which includes patients diagnosed with a malignancy (or a few specific benign disorders) before the age of 18 years between January 1, 1962, and December 31, 2001, alive 5-years postdiagnosis, and treated in one of the 7 Dutch pediatric oncology and stem cell transplant centers. Inclusion criteria for this study was CCSs currently alive, aged 18 years or older, and living in the Netherlands.

### Study Design

CCSs were randomly allocated to 1 of 3 study arms in a 1:1:1 ratio using computer software (nQuery version 7). Stratified sampling was used to achieve balanced representation of subgroups defined by gender and study center. The invitation strategies which were used in the different study arms combined 2 questionnaire modes and 2 reminder strategies (Table 1). In the first reminder strategy (study arms 1 and 2), 2 postal reminders and 1 telephone reminder followed the initial invitation, whereas in the second reminder strategy (study arm 3), only 1 postal reminder and 1 telephone reminder followed the initial invitation, with a period of 3 weeks between each reminder. Invitations and reminders were sent during September 2012 and June 2013. Questionnaires of CCSs were accepted until September 1, 2013, so that the study lasted exactly 1 year (International Standard Randomized Controlled Trial Number 84711754).

**Table 1:** Study arms.

	Study arm 1 n=249	Study arm 2 n=251	Study arm 3 n=250
Initial invitation	Web-based questionnaire	Web-based questionnaire	Web- and paper-based questionnaire
First reminder (after 3 weeks, in case of no response to initial contact)	Web-based questionnaire	Web- and paper-based questionnaire	Web based and option to apply for new paper-based questionnaire
Second reminder (after 3 weeks, in case of no response to 1st reminder)	Web- and paper-based questionnaire	Web-based and option to apply for new paper-based questionnaire	Telephone contact
Third reminder	Telephone contact	Telephone contact	

All CCSs received a postal package including a cover letter signed by the local physician responsible for CCSs follow-up care in which the login procedure for the Web-based questionnaire was explained and login details were given. In addition, an information sheet on the DCOG LATER study, an information sheet on the questionnaire study, an informed consent form, a refusal form for declining participation, and a prestamped return envelope were included. On the informed consent form an additional option was depicted to ask consent for linking data from the questionnaire to medical registries and information from the general practitioner (GP). On the refusal form, CCSs were asked if they could be contacted for a shortened telephone survey to ascertain baseline characteristics and health status and to ask consent for medical record release to collect information from a survivors’ GP or other treating physician and medical registries.

Paper-based questionnaires were added to the invitation at various time points depending on the study arm. CCSs either received the paper-based questionnaire at first contact (study arm 3), second contact (study arm 2), or third contact (study arm 1). All CCSs receiving the Web-based questionnaire at any of the time points had the option to apply for a copy of the paper-based questionnaire by contacting the study coordinator through email or telephone. The study coordinator also followed up the CCSs via phone calls (telephone reminders), which were performed at various time points (morning, afternoon, evening) and days. In case of a successful contact, CCSs were asked if they were willing to complete the questionnaire, either on paper or through the Internet. Survivors also had the option to complete a shortened telephone survey of 16 questions instead of completing the entire questionnaire. In case they indicated that they were not willing to participate, they were requested to return the refusal form.

If a survivor responded to the study invitation or one of the reminders, the survivor was considered a responder. If there was no response whatsoever, the survivor was considered a nonresponder. Responding CCSs could be further divided into participants and nonparticipants. A survivor was considered a participant when the paper- or Web-based questionnaire (with at least two thirds of the questions completed) was sent back. Nonparticipants included CCSs responding by answering the shortened telephone survey or by returning the refusal form with or without consent for medical record release.

CCSs who completed the telephone survey or returned the refusal form were asked about their reasons for not participating. CCSs had the option to choose between one or more of the following options: (1) I have already participated in many studies, (2) I do not want to be confronted with the past, (3) I think the questionnaire is too long, (4) I find the information about the study unclear, (5) I have no time to fill out the questionnaire, (6) I have no interest in this study, and (7) other reasons.

### Questionnaire

A paper- and Web-based questionnaire for CCSs were developed to collect information on general health and lifestyle. Both similarly collected information on education, socioeconomic status, medical history, disease symptoms, medication use, lifestyle, and quality of life. Different versions were used for male and female CCSs to account for differences in questions about reproduction and sexuality. The questionnaires for male and female contained 97 and 112 questions, respectively. The paper- and Web-based questionnaires were identical in number, type, wording, and order of questions posed. In the paper-based version, CCSs were explicitly instructed where they were allowed to skip questions that were not relevant to them (based on specific answers). In the Web-based version, these questions were automatically skipped. In general, it was possible for CCSs to leave questions open. Survivors had the option to save and log out of the Web-based questionnaire, and to log in again at another time. The time to complete the questionnaire was estimated to be 30 minutes.

To assess satisfaction of CCSs with the questionnaire, 4 questions were added to the standard questionnaire. The first question inquired the reason for choosing the paper-based instead of the Web-based questionnaire and vice versa. Furthermore, it was inquired whether any questions in the questionnaire had been difficult to answer and how much time it took to complete the questionnaire. Finally, CCSs were asked to indicate their agreement with 5 statements concerning their satisfaction with the questionnaire, which they had to answer on a Likert scale from 1 (strongly disagree) to 5 (strongly agree).

### Data Analysis

Data were analyzed using the statistical program R (version 2.15.1). Descriptive statistics were used to describe (1) differences between CCSs allocated to the different study arms, (2) response characteristics with respect to the different study arms, reminders, and questionnaire modes, (3) reasons for not participating, (4) participant characteristics in relation to questionnaire mode preference, and (5) participants satisfaction with the paper- and Web-based questionnaire. It was determined whether these differences were statistically significant using Kruskal-Wallis tests for continuous data and Pearson chi-square tests for categorical data. As the randomization to study arms was stratified by center, it was important to take the clustering of CCSs within centers into account [32]. Hence, for each Kruskal-Wallis and Pearson chi-square test, test statistics and associated degrees of freedom for each single center were calculated. The results were summed and *P* values were obtained using the summed test statistics and a chi-square distribution with the summed degrees of freedom [33]. *P* values less than .05 were regarded as statistically significant. However, for post hoc tests on all pairs of differences between the 3 arms of the study, the Bonferroni correction was implemented and *P* values less than .0167 (0.05/3) were regarded as statistically significant [34]. The agreement of CCSs to 5 different statements on a 5-point Likert scale were categorized into the following 3 categories: “Agree” with the statement (points 4 and 5), “Neutral” (point 3), or “Disagree” (points 1 and 2).

CCSs who completed the questionnaire were inquired on their highest achieved educational level. The International Standard Classification of Education (ISCED) was used to classify educational level [35]. The ISCED comprises 8 educational levels, which were further categorized to low (early childhood education, primary education, lower secondary education, ISCED levels 0-2), medium (upper secondary education, postsecondary nontertiary education, short-cycle tertiary education, ISCED levels 3-5), and high (bachelor or equivalent education, masters or equivalent education, doctoral or equivalent education, ISCED levels 6-8).

## Results

Overall, 750 survivors were randomly selected from a total of 2958 eligible adult CCSs from the 3 participating centers. The sociodemographic and treatment-related characteristics of the survivors in each of the 3 study arms are shown in Table 2. The randomization, stratified by gender and study center, resulted in representative cohorts in the study arms.

### Participation Rates per Study Arm

**Figure 1:**
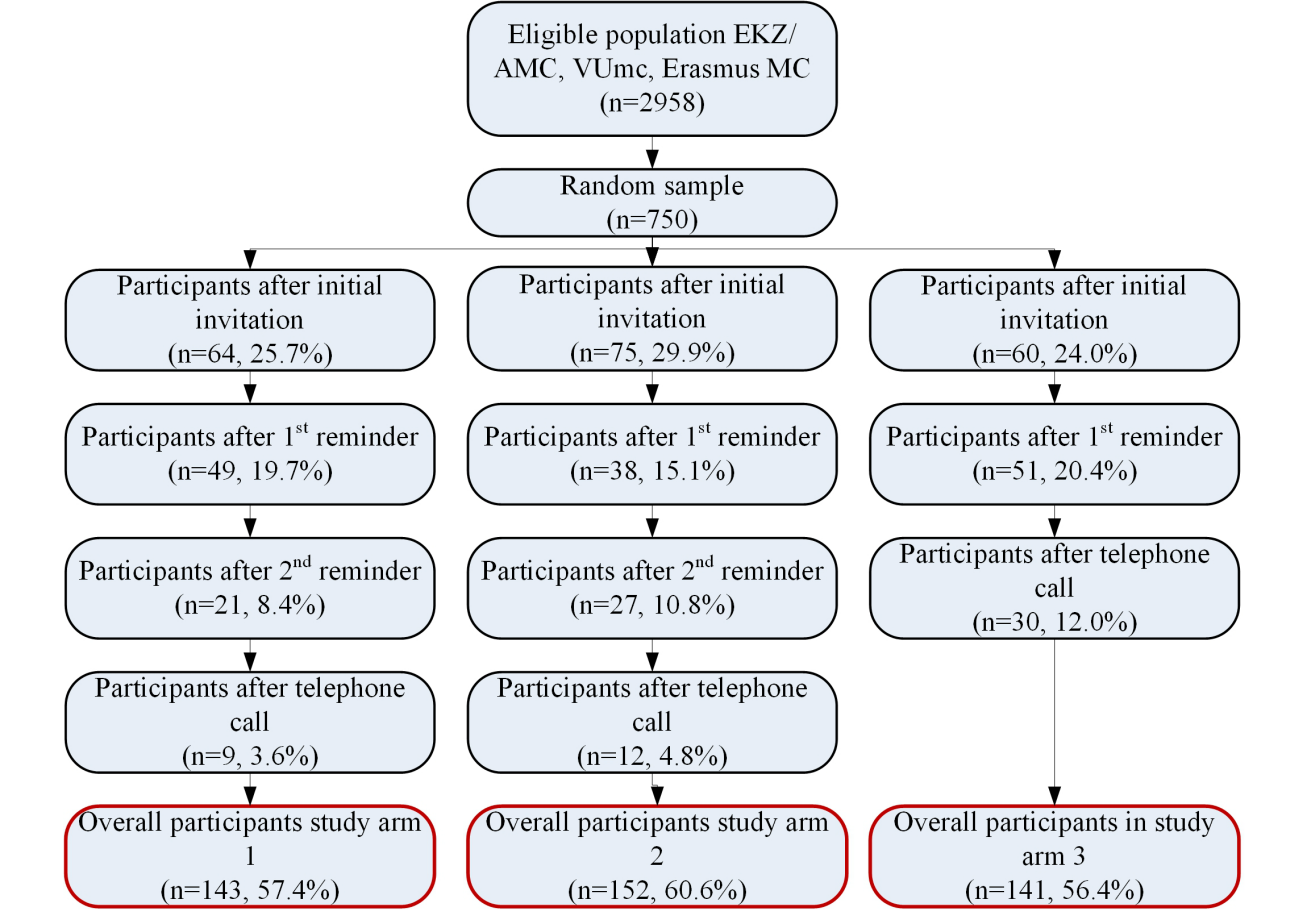
Flow diagram of the study.

The participation rates for all contact moments in each study arm are summarized in Figure 1. Overall, 58.1% of CCSs (436/750) completed the questionnaire, and participation rates were similar in all study arms with 57.4% (143/249) in study arm 1, 60.6% (152/251) in study arm 2, and 56.4% (141/250) in study arm 3 (*P*=.09).

In study arm 1, 64 (25.7%, 64/249) CCSs completed the Web-based questionnaire after the initial invitation. Nonresponding CCSs were reminded by a postal letter to complete the Web-based questionnaire, after which an additional 49 (19.7%, 49/249) completed the questionnaire. The second reminder consisted of a mixed-mode invitation (containing both the Web- and paper-based questionnaires). An additional number of 21 CCSs (8.4%, 21/249) completed the questionnaire after this reminder. A final telephone contact yielded an additional participation of 9 (3.6%, 9/249). Overall, 143 (57.4%, 143/249) CCSs completed the questionnaire, with 35 (14.1%, 35/249) completing the paper-based questionnaire and 108 (43.4%, 108/249) completing the Web-based questionnaire.

In study arm 2, 75 (29.9%, 75/251) CCSs completed the Web-based questionnaire after the initial invitation. Nonresponding CCSs received a mixed mode invitation as a first postal reminder. An additional 38 (15.1%, 38/251) CCSs completed the questionnaire after this reminder. Nonresponding CCSs received an additional second postal reminder following which 27 (10.8%, 27/251) CCSs completed the questionnaire. After a final telephone call, an additional 12 (4.8%, 12/251) CCSs completed the questionnaire. In total, 152 (60.6%, 152/251) CCSs completed the questionnaire, with 60 (23.9%, 60/251) CCSs completing the paper-based questionnaire and 92 (36.7%, 92/251) CCSs the Web-based questionnaire.

CCSs in study arm 3 initially received a mixed-mode invitation. After this invitation, 60 (24.0%, 60/250) CCSs completed the questionnaire. Nonresponding CCSs received a postal reminder, after which 51 more CCSs (20.4%, 51/250) completed the questionnaire. In addition, 30 (12.0%, 30/250) CCSs completed the questionnaire after telephone contact. A total number of 141 (56.4%, 141/250) CCSs participated, 101 (40.4%, 101/250) CCSs by completing the paper-based questionnaire and 40 (16.0%, 40/250) CCSs by completing the Web-based questionnaire.

In total, we attempted to contact 261 CCSs through a telephone reminder. Of these CCSs, we were unable to reach 67 CCSs (25.7%, 67/261) from all attempted reminders; and 38.5% (67/174) of CCSs remained nonresponders at the end of study. Approximately 40 hours were spent calling these CCSs (telephone reminder) with an average of 3 attempts per survivor (approximately 783 in total) and 3 minutes per attempt.

**Table 2:** Sociodemographic and treatment-related characteristics of the CCSs in each study arm.

Characteristic		Study arm 1	Study arm 2	Study arm 3
Number of survivors, n		249	251	250
Median current age (years), range		29 (18-58)	30 (18-61)	31 (18-60)
Median age at childhood cancer diagnosis (years), range		6 (0-17)	6 (0-17)	6 (0-17)
Median time since diagnosis (years), range		23 (10-46)	23 (10-49)	23 (10-49)
**Gender, n (%)**				
	Male	122 (49.0)	127 (50.6)	124 (49.6)
	Female	127 (51.0)	124 (49.4)	126 (50.4)
**Study center, n (%)**				
	EKZ/AMC	114 (45.8)	111 (44.2)	112 (44.8)
	Erasmus MC	80 (32.1)	83 (33.1)	82 (32.8)
	VUmc	55 (22.1)	57 (22.7)	56 (22.4)
**Diagnosis, n (%)**				
	Central nervous system tumor	31 (12.5)	38 (15.1)	38 (15.2)
	Leukemia	77 (30.9)	66 (26.3)	58 (23.2)
	Lymphoma	41 (16.5)	43 (17.2)	48 (19.2)
	Renal tumor	27 (10.8)	38 (15.1)	32 (12.8)
	Other	73 (29.3)	66 (26.3)	74 (29.6)
Chemotherapy, n (%)		199 (79.9)	187 (74.5)	191 (76.4)
Radiotherapy, n (%)		95 (38.2)	86 (34.3)	98 (39.2)
Surgery, n (%)		162 (65.1)	176 (70.1)	174 (69.6)
Other therapy, n (%)		135 (54.2)	129 (51.4)	128 (51.2)
Recurrence of disease, n (%)		39 (15.7)	30 (12.0)	31 (12.4)
Recent visit to late effect outpatient clinic (<2 years), n (%)		87 (34.9)	103 (41.0)	98 (39.2)

### Effect of Mixed-Mode Questionnaires on Participation Rates

At the initial invitation, the Web-only invitation group consisted of all CCSs in study arms 1 and 2, and the CCSs in study arm 3 received a mixed mode invitation. Although the proportion of participants after initial invitation was lowest in study arm 3, it was not significantly different compared to study arms 1 and 2 (24.0% and 27.8%, respectively, *P*=.55). To investigate the effect of adding a paper-based questionnaire to a postal reminder on participation rates, we compared the Web-only invitation group consisting of CCSs in study arm 1 with the mixed-mode invitation group consisting of CCSs in study arm 2. CCSs in study arm 3 were excluded from these analyses because they had already received the paper-based questionnaire with the initial invitation. Results show that the proportion of participants was not significantly different between the Web-only and mixed mode invitation group after the first postal reminder (19.7% in study arm 1 and 15.1% in study arm 2, *P*=.48). When receiving the mixed-mode invitation, in all study arms more CCSs preferred completing the paper-based questionnaire over the Web-based questionnaire (17.2% vs 6.8% in study arm 3, 10.4% vs 4.8% in study arm 2, and 5.2% vs 3.2% in study arm 1).

### Characteristics of Nonparticipants

The number of nonparticipants across all study arms was 140 CCSs (18.7%, 140/750). The number of nonparticipants was highest in study arm 1 (21.3%, 53/249) compared with 18.7% (47/251) and 16.0% (40/250) in study arms 2 and 3, respectively. The proportion of nonparticipants did not differ significantly across study arms. There were 29 (3.9%, 29/750) CCSs who did complete a short telephone questionnaire, 50 (6.7%, 50/750) CCSs who provided consent for medical record release only, and 61 (8.1%, 61/750) CCSs who refused participation altogether.

The reasons for nonparticipation are shown in Table 3. There were 2 main reasons for nonparticipation: CCSs did not want to be confronted with their past (26.4%, 37/140) and/or they indicated to have already participated in many other studies in the past (24.3%, 34/140). Another important reason was that CCSs found the questionnaire too long (10.7%, 15/140).

**Table 3:** Reasons for declining to complete the questionnaire (N=140).

Reasons	Telephone survey or refusal, n (%)
Unknown	18 (12.9)
I do not want to be confronted with the past	37 (26.4)
I have already participated in many studies	34 (24.3)
I think the questionnaire is too long	15 (10.7)
I have no time to fill out the questionnaire	11 (7.9)
I have no interest in this study	11 (7.9)
I have had bad experiences in the past with research/care	6 (4.3)
I am currently unable to fill out the questionnaire	5 (3.6)
The answers to the questions are already available at the clinic	4 (2.9)
I am unable to answer the questions due to a mental handicap	4 (2.9)
I find the information about the study unclear	0 (0.0)

### Questionnaire Mode Preferences

The differences in characteristics of the survivors completing the paper-based questionnaire compared to survivors completing the Web-based questionnaire are shown in Table 4. Gender, age at start of the study, age at diagnosis, years since diagnosis, follow-up center, diagnosis, treatment, marital status, and whether or not the survivor had recently visited a follow-up clinic did not have a significant effect on the survivors’ choice of questionnaire mode. CCSs who completed the paper-based questionnaire were more likely to be unemployed (20.9% vs 10.5%, *P=* .015) and lower educated (17.9% vs 7.1%, *P*=.008).

**Table 4:** Differences in participant characteristics between paper-based and web-based questionnaires.

		Paper-based questionnaire	Web-based questionnaire	*P* value
Survivors, n		196	240	
Median age, range		30 (18-61)	31 (18-57)	.73
Median age at diagnosis, range		6 (0-17)	6 (0-17)	.88
Median number of years since diagnosis, range		23 (10-49)	23 (11-45)	.52
**Gender, n (%)**				.96
	Male	93 (47.4)	109 (45.4)	
	Female	103 (52.6)	131 (54.6)	
**Follow-up center, n (%)**				
	AMC	104 (53.1)	104 (43.3)	
	Erasmus	50 (25.5)	84 (35.0)	
	VUmc	42 (21.4)	52 (21.7)	
**Diagnosis, n (%)**				.40
	Central nervous system tumor	26 (13.3)	24 (10.0)	
	Leukemia	50 (25.5)	57 (23.8)	
	Lymphoma	39 (19.9)	48 (20.0)	
	Renal tumor	29 (14.8)	38 (15.8)	
	Other	52 (26.5)	73 (30.4)	
Chemotherapy, n (%)		153 (78.1)	192 (80.0)	.91
Radiotherapy, n (%)		81 (41.3)	90 (37.5)	.40
Surgery, n (%)		133 (67.9)	173 (72.1)	.73
Other therapy, n (%)		104 (53.1)	124 (51.7)	.81
Recurrence of disease, n (%)		26 (13.3)	33 (13.8)	.70
Recent visit to late effect outpatient clinic (<2 years), n (%)		92 (46.9)	99 (41.2)	.76
**Marital status, n (%)**				.28
	Single	72 (36.7)	77 (32.1)	
	In a relationship	68 (34.7)	85 (35.4)	
	(Ever) Married	56 (28.6)	78 (32.5)	
**Employment status, n (%)**				.02
	Student	29 (15.2)	45 (18.8)	.83
	Employed	122 (63.9)	169 (70.7)	.24
	Unemployed	40 (20.9)	25 (10.5)	.015
	Unknown	5	1	
**Educational level, n (%)**				.001
	Low^a^	35 (17.9)	17 (7.1)	.008
	Medium^b^	107 (54.6)	135 (56.2)	.67
	High^c^	54 (27.6)	88 (36.7)	.04

^a^Low educational level: ISCED levels 0 to 2.

^b^Medium educational level: ISCED levels 3 to 5.

^c^High education level: ISCED levels 6 to 8.

There were 248 participating CCSs who had received a mixed-mode invitation with the initial invitation (56.9%, 141/248; study arm 3) or with one of the reminders (43.1%, 107/248; study arms 1 and 2) (Figure 1). Furthermore, 27 (3.6%, 27/750) CCSs who received a Web-only invitation requested the paper-based questionnaire from the study personnel; among these 27, 4 (14.8%) CCSs had also completed the Web-based questionnaire. A total of 275 CCSs thus had the choice to complete either the paper- or Web-based questionnaire. Of these, 79 (28.7%, 79/275) chose to complete the Web-based questionnaire compared to 196 (71.3%, 196/275) who chose the paper-based questionnaire. These CCSs were also asked about their reason for choosing the particular questionnaire mode (see Multimedia Appendix 1). The question was not answered by 8 CCSs (4 who completed the paper-based questionnaire and 4 who completed the Web-based questionnaire). For both paper- and Web-based questionnaires, the main reason was that CCSs found the questionnaire mode easier to use: 62.0% (119/192) of CCSs completed the paper-based questionnaire and 84% (63/75) completed the Web-based questionnaire. Other reasons for those CCSs who completed the paper-based questionnaire include practical reasons (14.6%, 28/192) and length of the questionnaire (9.4%, 18/192). For those CCSs who chose the Web-based questionnaire additional reasons were that they did not need to leave the house to go to the mailbox to send the questionnaire (25%, 19/75) and because of the length of the questionnaire (15%, 12/75).

### Questionnaire Satisfaction

Results show that, after correction for educational level, CCSs completing the Web-based questionnaire more often indicated that the questions were difficult to answer compared to CCSs completing the paper-based questionnaire (74.3% vs 62.8%, *P*=.02). Furthermore, CCSs spent more time (42.6 vs 37.7 minutes, *P*=.05) completing the Web-based questionnaire than the paper-based questionnaire. Although this trend was not statistically significant, when CCSs were asked whether they preferred to complete the other questionnaire mode next time, 18% (31/177) of participants of the paper-based questionnaire answered affirmatively, as compared to 10% (23/236) of participants of the Web-based questionnaire (*P*=.08). The proportion of survivors agreeing on statements assessing CCS’s satisfaction with the questionnaire for both questionnaire modes is summarized in Multimedia Appendix 2.

## Discussion

### Principal Findings

This is one of the first studies examining the influence of Web-based versus paper-based questionnaire on participation rates of CCS. Although the study had sufficient power to detect a difference of 15%, no differences in participation rates were found between the 3 study arms. The results also showed no difference in participation rates of CCSs who received an initial invitation for the Web-based questionnaire only versus CCSs who received an initial invitation to complete either a paper- or Web-based questionnaire. Furthermore, adding a paper-based questionnaire to a first postal reminder did also not result in higher participation rates compared to a reminder consisting of a Web-based questionnaire only.

In addition, when offered the choice to complete either the paper- or the Web-based questionnaire, most CCSs chose to complete the paper-based questionnaire. Furthermore, a significantly larger proportion of unemployed and/or low educated CCSs completed the paper-based questionnaire, although these results may have been influenced by different reminder strategies used in the different study arms. Most CCSs preferred the paper-based over the Web-based questionnaire as they considered the paper-based questionnaire more easy to use (60.7%, 119/192), yet the same reason was given by CCSs who completed the Web-based questionnaire (80%, 63/75). However, CCSs completing the Web-based questionnaire more often rated questions difficult to answer (74.3%, 176/237) compared to CCSs completing the paper-based questionnaire (62.8%, 118/188) and they also took on average 5 minutes more to complete the questionnaire.

### Comparisons With Other Studies

Previous questionnaire studies conducted among CCSs have yielded participation rates between 50% and 90% [12-19]. The overall participation rate in this study was within this range, and although we consider it rather low (58.1%, 436/750), it is in line with recent trends in epidemiological studies [11]. Decreases in participation rates in these types of studies can partly be explained by an increase of individuals explicitly declining participation. In this study, 140 invited CCSs (18.7%, 140/750) declined to complete the questionnaire, compared with about 5% in previous studies among CCSs [36,37]. An explanation for the increase in individuals declining participation is that there has been an increase in the number of requests to participate in scientific research for individuals over the past decades [11]. This increasing number of requests may become an intrusion on personal lives, limiting the willingness of individuals to participate. A quarter of the CCSs declining participation in this questionnaire study indicated that the reason for declining is that they had already participated in many other studies or that they did not wanted to be confronted with their past. It is conceivable that this leads to participation bias as evidence points out that individuals are much more likely to participate when the study concerns a topic which they consider of great importance to their lives [11]. As such, CCSs not suffering from severe late effects or having bad experiences with medical follow-up may be less inclined to participate in research on long-term effects, which would most certainly lead to an overestimation of the prevalence of late effects among the CCSs population [30]. Fortunately, in the Netherlands, obtaining information about nonresponders is allowed. As such, we are currently gathering data on health status and risk factors of nonresponding CCSs and CCSs that consented for medical record release by sending a questionnaire to their GP. This GP questionnaire will make it possible to compare outcome measures of the questionnaire among different response categories, except for CCSs declining participation and medical record release (8.1%, 61/750). Because data on nonparticipants are usually lacking, this will provide unique opportunities to measure and quantify selection bias.

Edwards et al [21] reviewed randomized controlled trials assessing methods to increase participation rates of paper-based and Web-based questionnaires. The probability of participation increased by more than a quarter with a follow-up contact after the initial invitation. In our study, we found a similar increase in participation rates with, on average, a 25% increase after the first postal reminder and an overall increase of 20% after the second postal and third telephone reminder. Although a combined strategy of postal and telephone reminders substantially improved CCSs participation rates, caution should be taken when interpreting results as different questionnaire modes were used within the study arms.

Previous studies showed ambiguous results regarding participants’ preferences for questionnaire modes. In a meta-analysis by Shih et al [23], higher participation rates of participants were found for paper-based than for Web-based questionnaires. However, no differences in participation rates were found between these questionnaire modes when offered in a mixed-mode invitation. In a recent study by Van den Berg et al [24], no differences were found in participation rates of female CCSs invited to complete either a paper- or a Web-based questionnaire. However, the CCSs who were invited through a paper-based questionnaire preferred completing the paper-based over the Web-based version. Our study confirms these results, although this study also showed that offering a paper-based questionnaire with a reminder contact ultimately does not influence the participation rate. In general, CCSs prefer to complete a study questionnaire on paper, even at a relatively young age, where a tendency toward preferring Web-based questionnaires was expected [11,27,29]. This may be explained by the fact that the questionnaire used in this study contained multiple questions on a medical history, requiring CCSs to take the questionnaire to their parents’ home for further inquiries; a paper-based version may be more suited for this purpose.

One concern with using multiple modes for data collection is the possibility that the results from different data collection modes are not comparable because participants across modes differ in certain characteristics [25,27,38]. It is thus important to investigate potential differences in participant characteristics opting for different questionnaire modes. Previous studies have shown that gender, age, educational level, and socioeconomic status can influence questionnaire mode preferences [11,24,25,27-29,38,39]. Participants of Web-based questionnaires more often are male, younger, higher educated, and employed. In this study, a lower proportion of unemployed and low educated CCSs completed the Web-based questionnaire compared with the paper-based questionnaire. One explanation for this finding may be that CCSs suffering from severe cognitive late effects, such as brain tumor or central nervous system-irradiated leukemia survivors, need help from a parent or other relative in completing the questionnaire. In 63% (32/51) of the low educated and 46% (30/65) of the unemployed survivors, a third party had indeed supported them in completing the questionnaire, whereas only 16% (62/380) of the higher educated and 18% (64/360) of the employed CCSs were assisted by a parent or relative. Hence, it seems that a paper-based questionnaire is more suitable when completing the questionnaire with help of other persons. Another explanation could be the lack of access of lower educated or unemployed CCSs to a computer or the Internet. In general, higher educated and employed people more often have access to the Internet at home than less educated, unemployed persons [40]. In this study, a higher proportion of low educated and unemployed CCSs from the Web-only group contacted the study office to request a paper-based questionnaire because they did not have regular access to a computer or the Internet. Of the participants, 11.9% (52/436) had a low education and 14.9% (65/436) is unemployed. There were 27 CCSs who had received an invitation to complete the Web-based questionnaire only and requested a paper-based questionnaire. Of those, 15% (5/27) completed the Web-based questionnaire anyway. Out of the 23 CCSs who completed the paper-based questionnaire, 26% (6/23) had a low education and 36% (8/23) were unemployed (4%, 1/23, unknown), which are higher than the percentages of low educated and unemployed in the participant group.

### Limitations

There are a number of factors limiting the generalizability of our study results to patient populations other than CCSs. First, the most ideal study design to evaluate the effectiveness of a combination of follow-up strategies and paper- versus Web-based questionnaires would have been a sequential multiple assignment randomized trial [41]. However, because our study was set up as a pilot study aiming to determine the most appropriate invitation strategy for the entire Dutch cohort of survivors, the decision for choosing the current certain study design was mostly based on practical considerations. By choosing a study design as mentioned above we would have to include even more study arms and a larger study group, thereby hampering the goal of this study to select the most appropriate strategy for the entire Dutch cohort.

Second, the CCSs population is a unique study population often confronted with (severe) long-term side effects, varying in need and type of follow-up care from other patient populations. Therefore, their involvement in research studies may differ from other patient populations. In addition, some CCSs in our study had already been frequently invited for scientific research in the past decade. This can be an advantage, because CCSs know what to expect, but it could also hamper the study as CCSs are less willing to participate due to the high frequency of such studies. The latter is an important issue for research groups that initiate nationwide late effects studies. Third, current CCSs represent a relatively young cohort, although the CCSs population will grow and age over the next decades. Fourth, this study was conducted among Dutch CCSs living in the Netherlands at the time of the study. Although Internet access at home is growing across European countries and the United States [42], the Netherlands is among the countries with the highest access rates [40]. This could have led to a relatively high proportion of participants that completed the Web-based questionnaire, making it difficult to generalize results to CCSs populations in other countries. Fifth, apart from an invitation to complete the questionnaire, CCSs were also asked to give consent to link questionnaire data with medical registries and GP information, which may potentially have influenced the participation rate.

CCSs represent a relatively young and mobile patient group [7], resulting in conceivably frequent changes in home address. Current addresses of CCSs in this study were all traced through the municipal registry system, but to trace CCSs telephone numbers we had to rely on data from the medical patient records from the participating long-term follow-up clinics. As such, we were unable to contact about one-third of the nonresponders by telephone because the telephone numbers appeared to be no longer in use (32%, 17/53 of nonresponders in study arm 1; 44%, 23/52 in study arm 2; and 38%, 26/69 in study arm 3).

Although we analyzed the effect of different questionnaire modes in combination with various follow-up strategies, there are other ways to improve participation rates of CCSs in questionnaire studies. For instance, it is known that prenotification of a study and incentives could further increase participates rates [43-45]. As participation rates may further decline in the coming years, future studies investigating other invitation and follow-up strategies to increase participation are of great importance.

### Conclusions

In this study, we found that invitation strategies offering a Web-based questionnaire without a paper-based questionnaire at the first invitation can be used without compromising participation rates of CCS. Research into invitation strategies that improve participation rates is important to limit the risk of selection bias and to increase statistical power. However, even if high participation rates are acquired, the results may still be subject to participation bias, as each invitation strategy has its own underlying self-selection mechanism. We showed that CCSs who were offered the choice between paper- and Web-based questionnaires preferred the paper-based questionnaire, especially those with lower education levels and being unemployed. Nevertheless, offering the choice between paper- and Web-based questionnaires will probably lead to the highest accrual participation rate. The results of this study are of great importance for gaining insight into selecting the best method for the accrual of CCSs in questionnaire-based studies and will be used to determine the strategy for the nationwide questionnaire survey of the DCOG LATER study. In further research, we will focus on investigating selection bias in the DCOG LATER questionnaire study.

## Appendix

Multimedia Appendix 1 Reasons for completing the paper- or Web-based questionnaire.

Multimedia Appendix 2 Proportion of CCS agreeing on statements regarding satisfaction with the questionnaire.

## Abbreviations

CCS: childhood cancer survivor
DCOG LATER: Dutch Childhood Oncology Group Late Effects Group
EKZ AMC: Emma Children’s Hospital Academic Medical Center
Erasmus MC: Erasmus Medical Center
GP: general practitioner
VUmc: Free University Medical Center
