# Supplementary material for: Effect of Web-Based Versus Paper-Based Questionnaires and Follow-Up Strategies on Participation Rates of Dutch Childhood Cancer Survivors: A Randomized Controlled Trial
Source: JMIR Cancer. 2015 Nov 24;1(2):e11. doi: 10.2196/cancer.3905 (PMC5367669; doi:10.2196/cancer.3905)
Supplement: Multimedia Appendix 1 [file cancer_v1i2e11_app1.pdf]

Multimedia Appendix 1. Reasons for completing paper-based or Web-based questionnaire.

	**Paper-based questionnaire N=192**	**Web-based questionnaire N=75**
For what reason did you decide to complete the paper/Web-based questionnaire instead of the Web/paper-based questionnaire? N (%)		
"I find it more easy to complete a questionnaire on paper/Internet"	119 (62.0)	63 (84.0)
"I do not have to leave the house to send the questionnaire"	NA	19 (25.3)
"I filled in the paper/Internet version for practical reasons"	28 (14.6)	11 (14.7)
"The length of the questionnaire is the reason for filling in the paper/Internet version"	18 (9.4)	12 (16.0)
"I received the paper-based version, therefore I decided I might as well complete this version"	15 (7.8)	NA
Other reasons	33 (17.2)	6 (8.0)
