# Supplementary material for: Effect of Web-Based Versus Paper-Based Questionnaires and Follow-Up Strategies on Participation Rates of Dutch Childhood Cancer Survivors: A Randomized Controlled Trial
Source: JMIR Cancer. 2015 Nov 24;1(2):e11. doi: 10.2196/cancer.3905 (PMC5367669; doi:10.2196/cancer.3905)
Supplement: Multimedia Appendix 2 [file cancer_v1i2e11_app2.pdf]

Multimedia Appendix 2. Proportion of CCS agreeing on statements regarding satisfaction with the questionnaire.

	**Paper-based questionnaire**	**Web-based questionnaire**	***P* value**
It took too much time to complete the questionnaire, n (%)	18 (9.4)	24 (10.2)	.96
The questions were clearly displayed, n (%)	160 (83.3)	199 (84.3)	.85
The questions were hard to understand, n (%)	12 (6.2)	8 (3.4)	.74
Overall, I'm satisfied with this questionnaire, n (%)	148 (80.4)	192 (81.4)	.87
If I had to complete the questionnaire again I would rather complete the other (Web vs. paper) questionnaire mode, n (%)	31 (17.5)	23 (9.7)	.08
